# Evaluating the Diagnostic Efficacy of Using Pooled Samples for Chronic Wasting Disease Testing and Surveillance

**DOI:** 10.3390/pathogens13121133

**Published:** 2024-12-21

**Authors:** Monica Hepker, Jianqiang Zhang, Vellareddy Anantharam, Anumantha G. Kanthasamy, Jue Yuan, Wenquan Zou, Rachel M. Ruden

**Affiliations:** 1 Department of Biomedical Sciences, Iowa State University, Ames, IA 50011, USA; mhepker@iastate.edu (M.H.); vellareddy.anantharam@uga.edu (V.A.); anumantha.kanthasamy@uga.edu (A.G.K.); 2 Department of Veterinary Diagnostic and Production Animal Medicine, College of Veterinary Medicine, Iowa State University, Ames, IA 50011, USA; jqzhang@iastate.edu; 3 Department of Physiology and Pharmacology, University of Georgia, Athens, GA 30602, USA; 4 Department of Pathology, Case Western Reserve University School of Medicine, Cleveland, OH 44106, USAwenquanzou@ncu.edu.cn (W.Z.); 5 Institute of Neurology, Jiangxi Academy of Clinical Medical Sciences, The First Affiliated Hospital, Jiangxi Medical College, Nanchang University, Nanchang 330006, China; 6 Wildlife Bureau, Iowa Department of Natural Resources, Ames, IA 50010, USA

**Keywords:** chronic wasting disease, CWD, white-tailed deer, RT-QuIC, ELISA, monitoring, surveillance, pooled testing

## Abstract

Disease monitoring informs the opportunities for intervention by natural resource agencies tasked with managing chronic wasting disease (CWD) in wild cervids. However, allocating funds toward testing can reduce those available for education, outreach, and disease reduction. Implementation of more efficient testing strategies can help meet both an expanding need by resource managers and a burgeoning demand from the hunting public in North America. Here, we evaluated the efficacy of pooled testing using the enzyme-linked immunosorbent assay (ELISA), the current screening test used by veterinary diagnostic laboratories in the United States, and real-time quaking-induced conversion (RT-QuIC), an amplification assay that is being evaluated by the U.S. Department of Agriculture but is not yet approved or commercially available. The samples used in this study consisted of medial retropharyngeal lymph nodes (RPLNs) routinely collected by the Iowa Department of Natural Resources during the 2019–2020 surveillance season. The test pools contained tissue from one positive deer diluted in tissue from an increasing number of undetected deer, with each individual contributing an equal tissue volume. ELISA remained positive with pooling thresholds of 1:1, 1:2, 1:4, and 1:9 at a standard volume of tissue homogenate, whereas RT-QuIC remained positive with pooling thresholds of 1:1, 1:2, 1:4, 1:9, 1:19, and 1:49 at a 0.02% tissue dilution. Our results suggest that pooled testing can reduce diagnostic costs multi-fold, and RT-QuIC can be a viable screening test compatible with current field collection standards.

## 1. Introduction

White-tailed deer (*Odocoileus virginianus*) are the most widely consumed free-ranging game species in North America [1]. They are also susceptible to chronic wasting disease (CWD), an emerging disease caused by a misfolded infectious prion protein that has been detected in wild cervids from 35 U.S. states and 4 Canadian provinces as of September 2024 [2]. This makes CWD unparalleled in its scale and scope to expose novel hosts, including people, through consumption of infected tissues.

Monitoring for CWD is conducted annually across North America with varying levels of rigor, both in areas known to have infected populations and those where it has not been detected yet. The most common method of testing white-tailed deer is to collect post-mortem samples of the medial retropharyngeal lymph node (RPLN), or obex when RPLN is unavailable, and submit those tissues for screening via enzyme-linked immunosorbent assay (ELISA) using antibodies specific for the pathogenic isoform of the prion protein, PrP^CWD^. Samples that test positive are termed “Initial Reactors” or “Suspects” depending on the laboratory and often confirmed using immunohistochemistry (IHC), a method that detects the PrP^CWD^ present in fixed tissue. Although deer are vulnerable to contracting this disease throughout the year, functionally, surveillance is often tightly linked to the hunting seasons for sample collection and management interventions.

As such, the seasonal influx of thousands of lymph nodes results in days- to weeks-long testing delays as samples are individually screened by ELISA. Sample pooling is a well-known practice in animal agriculture to test a group of animals by combining their individual contributions and then taking a representative subsample [3,4]. By testing multi-fold more animals using the same amount of reagent and assay run time, pooling often drives down the per head testing cost, which is notable, considering that diagnostics were the second highest CWD-related expenditure reported by natural resource agencies behind staff time [5]. Alleviating this constraint can allow resource managers to allocate more funding dollars toward intervention, outreach, and education to better excise CWD at the root and switch from a reactive to proactive posture in areas where disease has not yet been found. Lower costs and shorter turnaround times may also make consumer-driven testing more attractive.

Previous studies found similar sensitivity and specificity between seed amplification assays (SAAs) like real-time quaking-induced conversion (RT-QuIC) and ELISA [6,7,8,9,10,11]. Castilla et al. (2005) also evaluated tissue homogenates from CWD-positive animals diluted with specific proportions of homogenates from test-negative animals [12]. While these studies were not explicitly designed to validate the accuracy of detection of a positive individual within a group of negative individuals, they effectively demonstrated that the concept would likely be successful. However, to date, no studies have explored the pooling thresholds for lymph tissue routinely collected by state agencies for CWD surveillance.

Here, we evaluate the performance of both the current screening test, ELISA, and an ultra-sensitive assay, RT-QuIC, under pooled testing conditions. RT-QuIC is designed to not just detect prion aggregates but amplify the aggregates themselves for enhanced pathogen detection. Therefore, we expect it to detect signatures of disease that fall below the threshold of detection by IHC, the current diagnostic “gold standard”. In accordance, Henderson et al. (2020) found that detection by RT-QuIC preceded that by IHC by months on serial biopsy of tonsil and rectoanal mucosa-associated lymphoid tissue [13]. Moreover, Burgener et al. (2022) found that IHC had a false negative rate in excess of 13% compared to RT-QuIC on RPLN, which can be more consequential to the end user than a false positive [14].

Currently, the Iowa Department of Natural Resources (DNR) screens roughly 5% of the deer harvested in Iowa each year for CWD. As the number of affected counties grows, hot spots expand, and venison consumers take more agency over making informed consumption decisions, we anticipate the demand for CWD testing for personal use to massively expand. Pooling samples will help veterinary diagnostic laboratories scale up their services to meet this burgeoning need and drive down costs to provide more equitable access to testing. It also creates an opportunity for amplification assays like RT-QuIC to become commercially viable, capitalizing on their superior sensitivity to achieve higher pooling thresholds and earlier detection of asymptomatic deer.

## 2. Materials and Methods

### 2.1. Tissue Collection and Diagnostic Testing

Throughout the 2019/2020 surveillance season (1 April 2019–31 March 2020), paired medial retropharyngeal lymph nodes (RPLNs) were routinely collected from wild white-tailed deer by trained Iowa DNR field staff and partners. Briefly, the head was reflected back using a single horizontal cut about one inch behind the lower jaw. The lymph nodes were then exteriorized and placed in individual Whirl-Pak bags with sample ID numbers linking them to metadata related to each deer.

One RPLN was designated the “A” sample and submitted for diagnostic testing at either the Iowa State University Veterinary Diagnostic Laboratory (ISU VDL) in Ames, IA, or the Colorado State Veterinary Diagnostic Laboratories (CSU VDL) in Fort Collins, CO. Lymph nodes were screened using the commercially available ELISA targeting PrP^CWD^ (TeSeE Purification and Detection Kits, Bio-Rad Laboratories, Inc., Hercules, CA, USA), following the protocols from the manufacturer. Samples with optical densities (ODs) below the plate-specific threshold of 0.035 plus the mean of the negative controls were reported as “Not Detected” or functionally test-negative. Any samples with ODs at or above this threshold were reported as “Initial Reactors” or “Suspects” and were then confirmed positive by the National Veterinary Services Laboratories in Ames, IA, or the CSU VDL using IHC targeting PrP^CWD^. On seldom occasion, ELISA-positive samples were not confirmed on IHC, and the other RPLN (designated the “B” sample) was submitted for IHC. If neither RPLN could be confirmed on IHC, the sample was deemed “Not Detected”. All other “B” samples were retained frozen in a standard −20 °C freezer.

### 2.2. Assigning Tissue Pools

Samples from 120 deer were initially included in this study based on the performance of their “A” sample (26 positive, 94 test-negative). Of note, all deer testing “Not Detected” (test-negative) originated outside a 20-mile buffer area of CWD deer management zone boundaries established following CWD detections in captive or wild deer. All “B” RPLNs were then screened individually in duplicate by ELISA and quadruplicate by RT-QuIC, with any samples showing disparate results excluded from pooling (Table 1). The tissue pools comprised one positive “spike” sample diluted in a variable number of samples from test-negative deer. Ultimately, 17 positive RPLNs and 78 test-negative RPLNs were used to create 39 tissue pools for evaluation by ELISA, including 6 at 1:1, 10 at 1:2, 6 at 1:4, and 17 at 1:9 pooling thresholds, and 63 tissue pools for evaluation by RT-QuIC using 0.02% tissue homogenate, including 11 at 1:1, 17 at 1:2, 6 at 1:4, 17 at 1:9, 6 at 1:19, and 6 at 1:49 pooling thresholds (see Supplementary Data).

### 2.3. Tissue Preparation for ELISA

Grinding tubes (Bio-Rad Laboratories, Inc., Hercules, CA, USA) were provided by ISU VDL to homogenize 200 ± 20 mg of tissue. For each pool, RPLNs were partially thawed and trimmed of excess connective tissue before being apportioned into the tared grinding tube using a scalpel. Importantly, as the pooling threshold increased, each RPLN contributed a smaller proportion of tissue ranging from a maximum of 100 ± 10 mg for the 1:1 dilution to a minimum of 20 ± 2 mg for the 1:9 dilution. We limited pool size to a maximum of 10 individuals (20 mg per individual) to keep human error within 10%. Individually labeled tubes were returned to ISU-VDL for routine processing and testing using USDA-approved protocols.

### 2.4. Tissue Preparation and Sample Processing for RT-QuIC

RPLNs were partially thawed and trimmed of excess connective tissue. Next, 50 ± 5 mg of tissue was cut and weighed into a 1.5 mL screw-cap Rino brand grinding tube (Next Advance, Inc., Troy, NY, USA) filled with ~100 mg of 0.5–1 mm zirconium oxide grinding beads. Sterile filtered 1 x phosphate-buffered saline (PBS) was added to each grinding tube to create a 10% weight/volume solution, and the tissue was homogenized at maximum speed (Bullet Blender 5 mL with adaptors for 1.5 mL screw-cap tubes; Next Advance, Inc., Troy, NY, USA) for 10 min. The resulting 10% homogenate was pipetted into a 2 mL tube for storage at −80 °C until evaluation by RT-QuIC.

The RT-QuIC assay was performed as previously described in Kondru et al. (2017) [15]. Briefly, individual samples were diluted to a concentration of 0.02% in a solution of 1 x PBS plus 0.05% sodium dodecyl sulfate (SDS). Next, 5 µL of the diluted sample was added to 95 µL of reaction mixture (350 mM NaCl, 0.1 mM EDTA, 10 μM Thioflavin T(ThT), 0.1 mg/mL Syrian Hamster (90–231) rPrP, and 0.0025% SDS in 1 x PBS at final concentration) in a black-wall, clear-bottom, 96-well plate. The plate was sealed and incubated on a shaking plate reader at 42 °C with 1 min cycles of double orbital shaking at 400 rpm followed by a 1 min pause. ThT fluorescence readings (450 ± 15 nm excitation and 480 ± 10 nm emission) were taken every 45 min for 30 h with a 24 h assay cut-off time. The fluorescence threshold was set as the average of the first six readings for all experimental sample wells (runtime 0:45–4:30 h) plus five times the standard deviation, and at least two of the four replicate wells needed to cross the threshold by 24 h for the sample to be considered positive.

For pooling studies, equal volumes of the 10% stock homogenate of each individual sample were combined, diluted, and 5 µL of the final dilution was applied to the assay. All samples, whether run individually or in pools, were tested in quadruplicate, with results reported as the group mean.

### 2.5. Data Analysis

The results from ELISA (OD) and RT-QuIC (mean time to threshold fluorescence) were analyzed with linear mixed-effects models in R, version 4.4.2, using the lme4 package [16,17]. The models included pooling threshold as the fixed effect (ELISA: pool sizes 1, 2, 3, 5, and 10; RT-QuIC: pool sizes 1, 2, 3, 5, 10, 20, and 50) and positive deer ID and number of replicates as crossed random effects. Of note, the values used for pool sizes of 1 originated from the pre-screening data, in which B-RPLNs were tested individually by ELISA (based on the duplicate mean) and RT-QuIC (based on the quadruplicate mean). GraphPad Prism version 8.0.1 for Windows was also used to generate some figures.

## 3. Results

### 3.1. Assay Agreement for Pools Up to 10 Individuals

**Figure 1:**
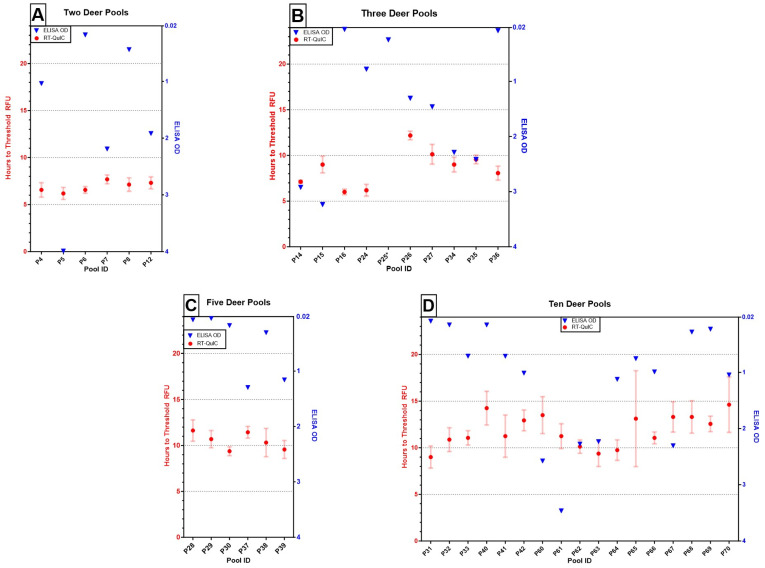
Results of ELISA (OD of single well, blue triangles) and RT-QuIC (mean time to threshold fluorescence of quadruplicate wells ± one standard error, red circles with bars) testing on pooling thresholds of (**A**) two deer (e.g., 1:1; *n* = 6), (**B**) three deer (e.g., 1:2, *n* = 10), (**C**) five deer (e.g., 1:4; *n* = 6), and (**D**) 10 deer (e.g., 1:9; *n* = 17). All pools across assays tested positive, except for P25, which failed to amplify within 24 h on RT-QuIC and is denoted with an asterisk (*).

Of the 39 pools evaluated by both assays, ELISA detected the positive in all pools, and RT-QuIC failed to detect the positive in one pool containing three deer within 24 h (P25, Figure 1B). The overall agreement between assays was 97.4% (38/39).

**Figure 2:**
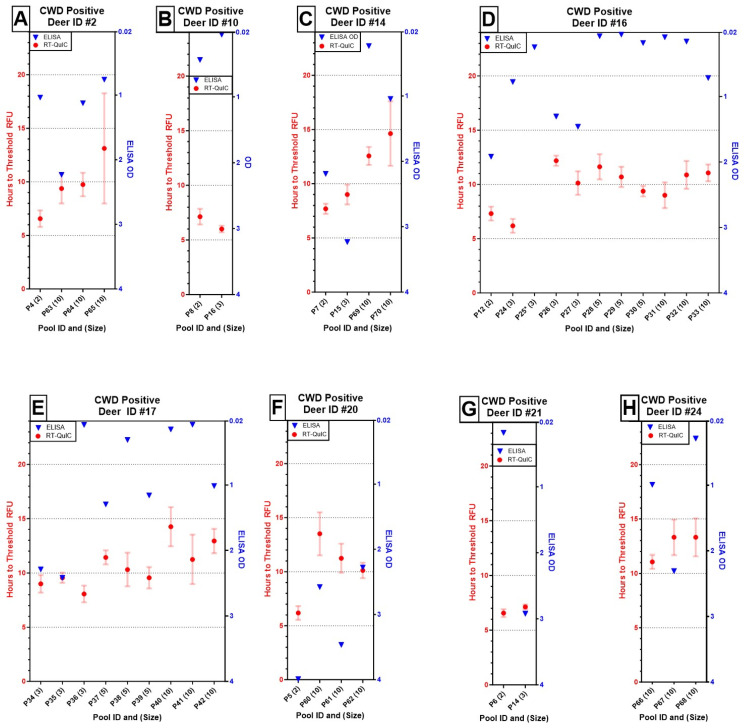
Results of ELISA (OD of single well, blue triangles) and RT-QuIC (mean time to threshold fluorescence of quadruplicate wells ± one standard error, red circles with bars) for eight positive deer at varied pooling thresholds indicated in parentheses next to the pool ID (**A**–**H**). Although Deer ID #16 (**D**) failed to amplify in one out of four RT-QuIC replicates at a pool size of three (*), it remained otherwise detectable up to a pool size of 10. Overall, OD values across individuals, regardless of pool size, were unpredictable from run to run. In contrast, the times to reach threshold fluorescence increased with pool size and remained fairly repeatable across pool size replicates by individual.

We also evaluated the consistency of detecting known positive deer against unique combinations of test-negative deer across the pooling thresholds and repeatability at the same pooling threshold (Figure 2). ODs fluctuated widely across positive deer IDs and, notably, across pool size replicates, whereas the time to cross threshold fluorescence showed less variable outputs at both levels.

### 3.2. Pools Exceeding 10 Individuals

**Figure 3:**
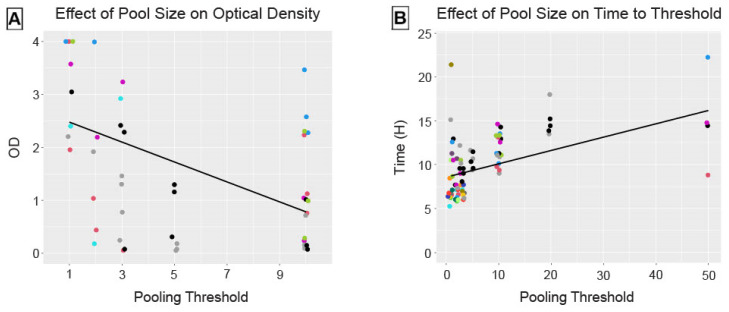
Pool size had a significant fixed effect on assay performance, decreasing OD on ELISA (**A**) and increasing time, in hours, to reach threshold fluorescence on RT-QuIC (**B**). The solid lines reflect the fixed effect estimates for pool size from the linear mixed-effects models. Points are colored by positive deer ID and reflect outputs from single wells on ELISA (duplicate mean from pre-screening used for pool size of 1) and the means of quadruplicate wells on RT-QuIC.

RT-QuIC was further used to screen pools of 20 and 50 individual deer. While we did observe a delay in the length of time to exceed threshold fluorescence, five of the six pools of 20 deer (83%%) and four of the six pools of 50 deer (67%) were detected before the 24 h assay cut-off time (Figure 3B). The remaining pools failed to amplify at least two replicate wells by 24 h (P49, P54, and P58; see Supplementary Data).

### 3.3. Effect of Pool Size on Assay Performance

Pool size had a significant negative linear effect (estimate = −0.188, 95% confidence interval (CI) [−0.259, −0.112]) on optical densities for ELISA (Figure 3A). Pool size had a significant positive linear effect (estimate = 0.152, 95% CI [0.096, 0.211]) on time, in hours, to reach threshold fluorescence for RT-QuIC (Figure 3B). This inverse relationship is as expected, given the different parameters reported by these two assays.

Although we evaluated positive deer ID and number of replicates as crossed random effects, the final models only contained positive deer ID based on a smaller Akaike information criterion (ΔAIC = 2) when the second parameter was dropped. The model containing only a random effect for the number of replicates performed significantly worse, or explained much less of the observed variation in optical density, than either model for ELISA (ΔAIC > 14). Although this model performed only slightly worse than including the crossed random effects for RT-QuIC (ΔAIC = 1.58), it explained significantly less variation in the time to threshold fluorescence than positive deer ID alone (ΔAIC = 3.58).

### 3.4. Improving RT-QuIC Performance

**Figure 4:**
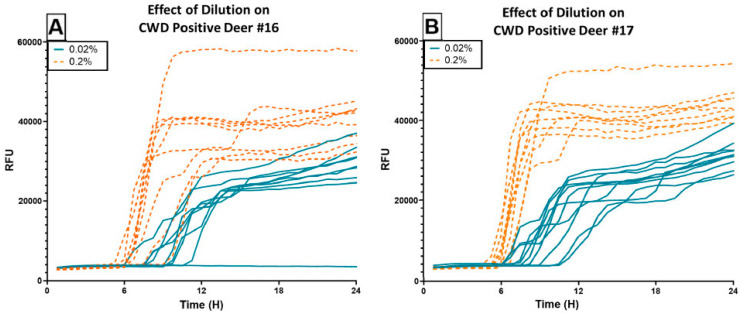
RT-QuIC results of using CWD Positive Deer #16 (**A**) and #17 (**B**) to spike pools of varying sizes, with samples applied to the assay at two different concentrations. The blue lines indicate sample performance when run at the standard dilution of 0.02%. The orange dotted lines indicate sample performance of the same pools run at 0.2%. Using the higher concentration appears advantageous for detection speed and overall fluorescence intensity.

A subset of 30 pools (6 per pooling threshold; see Supplementary Data) was run at 0.2% tissue homogenate, a 10× more concentrated sample application than standard protocol. We compared the performance of the 0.02% pools versus 0.2% pools and found that all pools run at the higher concentration exhibited a distinct leftward (earlier in hours) and upward (greater relative fluorescent units, RFU) detection character (Figure 4). Additionally, although P25 (1:2) amplified only one well at 29.25 h at the 0.02% dilution, well after the 24 h assay cut-off time, P25′ amplified all wells with a mean time to threshold fluorescence of 6.75 h at the 0.2% dilution. Likewise, P49′ (1:19), P54′ (1:49), P58′ (1:49) amplified all wells with mean times of 7.125 h, 7.875 h, and 9.188 h, respectively.

### 3.5. Blinded External Validation of Pooled Testing with RT-QuIC

**Figure 5:**
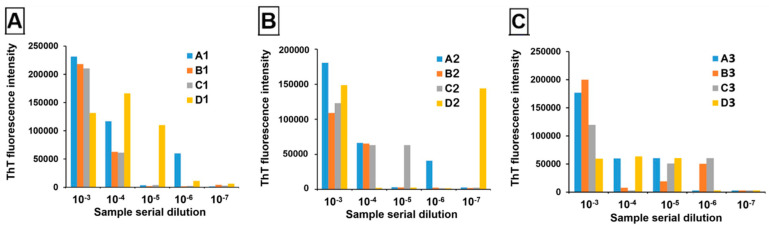
RT-QuIC results of serially diluting tissue from three positive deer, 1 (**A**), 2 (**B**) and 3 (**C**), at four pool sizes (A = 3 deer, B = 5 deer, C = 10 deer, D = 20 deer). ThT fluorescence intensity is presented as an average across quadruplicate wells, with samples exceeding 100,000 RFUs indicating that ≥2/4 wells amplified.

Twelve pooled samples at 0.02% tissue homogenate were tested blindly by the Zou laboratory at Case Western Reserve University School of Medicine. Samples comprised triplicate tissue pools containing unique positive deer (1–3) at four pooling thresholds (1:2 (A), 1:4 (B), 1:9 (C), and 1:19 (D)). These were then serially diluted out to 10^−7^ and evaluated in quadruplicate using RT-QuIC with slight modifications in protocol based on Orrù et al. (2017) [18] (Figure 5). All samples amplified in at least two replicate wells at 10^−3^ dilution, except for D3, meaning that even at a 1:190 dilution, two out of three positive deer remained detectable.

## 4. Discussion

In this study, we evaluated the feasibility of detecting tissue from a CWD-positive deer when diluted in a pool of test-negative individuals using both the currently validated screening test, ELISA, and an ultra-sensitive amplification assay, RT-QuIC. We ran these assays under idealized conditions, meaning all tissues originated from deer of known disease status, and most pools were spiked with strong, consistent positives. We were able to reliably detect positive deer in pools of up to 10 (1:9) using 20 mg of tissue per individual with ELISA and pools of up to 50 (1:49) using a 0.02% tissue homogenate per individual with RT-QuIC.

Although ELISA performed well at the tested pool sizes, screening larger pools may not be practical or prudent. Individual samples must be cut and weighed within a realistic margin of error, typically plus or minus 10% or 2 mg for pools of 10, which requires the use of highly precise instruments. An alternative approach may be grinding individual tissue homogenates first at maximum tissue volume (200 mg) and then pooling prior to loading onto the ELISA plate. This could reduce the likelihood of missing positive follicles from individuals with variable PrP^CWD^ deposition, either due to disease stage or host or strain factors [19,20,21]. That being said, the risk of ELISA returning false negatives given its inherent limit of detection, even under current screening practices, could be exacerbated by sample pooling, especially given the negative linear relationship we observed between pool size and optical density even with strong positives.

Yet, the greater challenge revealed in this study may be the lack of repeatability in the reaction characteristics with ELISA. ELISA outputs are given as an OD reading between 0 and <4 with the positive detection threshold set at 0.035 ± the average of the plate-specific negative controls. Although positive deer retained repeatable positive detection character, pools containing specific positive individuals did not result in predictable or repeatable ODs. For example, Deer ID #17 was successfully detected across nine pools containing either three deer (OD range: 0.078–2.415), five deer (OD range: 0.309–1.295), or ten deer each (OD range: 0.075–0.149). The range in OD did narrow with pool size, but this deer returned a weak reactor even when diluted with fewer deer (P36, Figure 2E). Given this variability in performance of strong positives, further study is warranted to understand how the pooling threshold identified here holds up under real-world conditions with positives of lower intensity.

In contrast, on RT-QuIC, positive deer achieved more consistent outcomes across replicates while supporting a positive linear relationship between pool size and the length of time to reach threshold up to pool sizes of 50. This result is consistent with several published studies that show that serially diluting individual positive tissue samples results in a negative linear relationship between the dilution factor and the time to detection [22,23]. Moreover, by using a ten-fold more concentrated tissue homogenate, we observed a shorter time to detection and higher endpoint RFU. Fluorescence curves that achieve earlier logarithmic growth with a more defined shoulder allow for easier interpretation of positivity (minimum of 2/4 replicates) versus random aggregation of the protein matrix that may occur with extended run times (e.g., >24 h). The improved performance of the more concentrated homogenate also suggests that lymphoid samples may be less subject to aggregation inhibition than samples originating from the central nervous system [24]. As such, concentrating the potential seed source, either by applying less diluent or coupling with capture techniques like iron oxide magnetic extraction, could improve the sensitivity and specificity of this assay [25].

By using two criteria to determine positivity, we lost some sensitivity in identifying positive pools P49 and P54, both of which amplified second and third replicate wells between 24 h, our assay cut-off time, and 30 h, when the assay was discontinued. In a real-world application, divergence from the negative control at these extended time points should still trigger retesting, either as a pool or individually, via RT-QuIC or ELISA out of an abundance of caution. However, we also maintained high specificity in rejecting negative pool P43, which amplified only one well. Autopolymerization, or random aggregation of the protein matrix in the absence of a positive seed (e.g., positive deer tissue), is a concern with using RT-QuIC, as it can lead to misclassifications and false positives. Nevertheless, true random aggregation would be unlikely to affect more than one replicate well of a sample, and therefore, adhering to minimum well count criteria can limit reporting errors. This same logic can also protect against human error while plating, where a microdroplet of homogenate from an adjacent well can seed an unexpected aggregation event. Given that even the sample pools that failed (P25 and P58) at the standard sample dilution of 0.02% performed as expected at 0.2%, using these criteria for positivity in concert with more concentrated homogenate may offer the greatest testing accuracy.

Taken together, the results of running both assays on the same tissue pools suggest that RT-QuIC produces a more consistent output across pool sizes with less intra-pool variation across replicates than ELISA. This raises an important consideration. We used the OD of the “B” RPLNs to infer the relative strength of the positives used in this study, for which there is some evidence of positive correlation [11]. However, whereas ELISA measures the availability of PrP^CWD^ antigen to bind the antibody, SAAs like RT-QuIC measure function, specifically the capacity for conversion and aggregation events within an available protein substrate [16]. As such, it may be misguided to use one assay to interpret the performance of another. At least at the lower pooling thresholds (≤10 deer), it appears that seeding activity is fairly insensitive to dilution effects (Figure 2). Moreover, despite working with a quarter of the tissue weight used in ELISA (50 mg versus 200 mg, though considerably less could be used to achieve 10% solution), RT-QuIC maintained a consistent signal, suggesting that seeding activity throughout the tissue is less variable than antigen, perhaps even when lymphoid follicles are absent [13]. Understanding how this assay performs with weaker positives, as characterized by ELISA, will be of utmost interest in marketing RT-QuIC on pooled samples as a viable screening approach.

Although the objective of this study was to evaluate the diagnostic accuracy of testing pooled samples compared to current screening practices, it is worth revisiting the data presented in Table 1. We excluded samples from the pooling experiments if the B-RPLN returned discordant results on ELISA or RT-QuIC with the disease status previously established by ELISA and IHC of the A-RPLN. This re-screening procedure was included given previous work by Bloodgood et al. 2021 indicating that ~6% of deer may test IHC-positive in one RPLN but not its pair [21]. It was also intended to minimize uncertainty while identifying pooling thresholds under controlled conditions. Interestingly, nine B-RPLNs (4/94 on the first test and 5/94 on the second test with no overlap in deer ID) came back as false positives, and two (1/26 on the first test and 2/26 on the second test with one overlapping sample) came back as false negatives on ELISA. By comparison, no B-RPLNs came back as false positives, but three (3/26) came back as false negatives on RT-QuIC, two of which were the false negatives on ELISA. Given these disparate results across lymph node pairs, it would be prudent to include tissue from A- and B-RPLNs at initial screening. Although this would functionally reduce the number of deer that could be screened at a given pool size, it would increase end-user confidence and, likely, diagnostic accuracy.

Yet, there are also the regulatory concerns of using an assay that has not yet been approved by the USDA as an official CWD test [26,27]. While this will remain a constraint in the application of amplification assays on farmed cervids, which must comply with the Chronic Wasting Disease Program Standards for the lawful interstate, and often intrastate, movement of individuals, testing of wild cervids is not subject to this regulatory oversight (see page 34 for comment on ELISA) [28]. Some natural resource agencies already deviate from the approved testing standards, screening samples with ELISA but not confirming those test-positive samples with IHC in areas where CWD is already considered endemic (R. Ruden, personal communication, 12 November 2024). We envision that a testing pipeline that includes sample pooling (whether using ELISA or RT-QuIC) would still involve confirmatory testing with ELISA and/or IHC. Samples from pools that flagged positive would be tested individually by ELISA. Test-positive samples could then be sent on for confirmatory testing by IHC, or the ELISA result could serve as the final disease status depending on the preference of the end user. In a future when amplification assays have received USDA approval, perhaps confirmatory testing would involve individual testing via RT-QuIC as a new “gold standard”. However, in the interim, while these assays remain in regulatory limbo, the pipeline we describe could be sound strategy for near immediate implementation at the veterinary diagnostic laboratories within the National Animal Health Laboratory Network that are already approved to run CWD testing [28].

Improving diagnostic testing for CWD was identified by the North American Interdisciplinary Chronic Wasting Disease Consortium as a priority need in 2019 [29]. A component of this certainly involves the type of test, such as SAAs, that may redefine the gold standard, but it also relates to how we implement the diagnostic assays currently available. With myriad natural resource agencies using ELISA as their screening test, the findings from this study have immediate relevancy and application depending on risk tolerance. Improving the time and cost effectiveness of the testing process is integral to sustaining CWD management and response activities as we reckon with managing a cervid resource within an expanding CWD geography. Pooled testing will not only improve throughput at veterinary diagnostic laboratories, which become natural bottlenecks in the testing process, but allow deer managers and one health practitioners to accommodate an expanding need for testing from hunters, as well as other end users like food banks, sportsmen’s groups, or meat processors participating in venison donation programs. It also offers a scalable solution. Although we identified pools of 10 individuals on ELISA and 50 individuals on RT-QuIC as viable thresholds under idealized conditions, in practice, pool size may be better optimized according to local or regional prevalence to minimize the number of positive pools requiring retest. We also need to better understand how pooling of individuals in early disease may impact the sensitivity of these assays, which may inform optimal pool sizes in areas that are apparently CWD-free. Nonetheless, this study offers the foundation for an alternative testing strategy that can help alleviate the diagnostic burden CWD poses in North America.

## Figures and Tables

**Table 1:** Lymphoid tissues from 120 individual white-tailed deer were evaluated for inclusion in this pooling study based on previous CWD testing of their “A” RPLN. “B” RPLNs were then screened for CWD using ELISA in duplicate and RT-QuIC in quadruplicate, and any individuals with discordant results across paired lymph nodes or B replicates were excluded from test pools.

	A-RPLN ELISA ± IHC	B-RPLN ELISA	B-RPLN RT-QuIC
CWD+	26	33	23
CWD−	94	87	97
False+		9	0
False−		2	3
Sensitivity		92.3%	88.5%
Specificity		90.4%	100.00%

## Data Availability

The original contributions presented in this study are included in the article/Supplementary Material.

## Supplementary Materials

The following supporting information can be downloaded at: https://www.mdpi.com/article/10.3390/pathogens13121133/s1, Data File S1: Supplementary Data_Testing Outcomes for Sample Pools, Data File S2: Pooling data_final, and R Script: Pooling Data_LMER_final.
